# Therapeutic strategies and potential implications of silver nanoparticles in the management of skin cancer

**DOI:** 10.1515/ntrev-2020-0117

**Published:** 2020-12-31

**Authors:** Shaloam Dasari, Clement G. Yedjou, Robert T. Brodell, Allison R. Cruse, Paul B. Tchounwou

**Affiliations:** Department of Biology, Environmental Toxicology Research Laboratory, NIH-RCMI Center for Environmental Health, Jackson State University, Jackson, MS 39217, United States of America; Department of Biological Sciences, College of Science and Technology, Florida Agricultural and Mechanical University, 1610 S. Martin Luther King Blvd, Tallahassee, FL 32307, United States of America; Department of Dermatology, University of Mississippi Medical Center, 2500N. State Street, Jackson, MS 39216, United States of America; Department of Dermatology, University of Mississippi Medical Center, 2500N. State Street, Jackson, MS 39216, United States of America; Department of Biology, Environmental Toxicology Research Laboratory, NIH-RCMI Center for Environmental Health, Jackson State University, Jackson, MS 39217, United States of America

**Keywords:** skin cancer, basal cell carcinoma, squamous cell carcinoma, malignant melanoma, biology, epidemiology, clinical manifestations, treatment, silver nanoparticles

## Abstract

Skin cancer (SC) is the most common carcinoma affecting 3 million people annually in the United States and millions of people worldwide. It is classified as melanoma SC (MSC) and non-melanoma SC (NMSC). NMSC represents approximately 80% of SC and includes squamous cell carcinoma and basal cell carcinoma. MSC, however, has a higher mortality rate than SC because of its ability to metastasize. SC is a major health problem in the United States with significant morbidity and mortality in the Caucasian population. Treatment options for SC include cryotherapy, excisional surgery, Mohs surgery, curettage and electrodessication, radiation therapy, photodynamic therapy, immunotherapy, and chemotherapy. Treatment is chosen based on the type of SC and the potential for side effects. Novel targeted therapies are being used with increased frequency for large tumors and for metastatic disease. A scoping literature search on PubMed, Google Scholar, and Cancer Registry websites revealed that traditional chemotherapeutic drugs have little effect against SC after the cancer has metastasized. Following an overview of SC biology, epidemiology, and treatment options, this review focuses on the mechanisms of advanced technologies that use silver nanoparticles in SC treatment regimens.

## 1 Introduction

Skin cancer (SC) incidence has been rapidly increasing in the United States for the past three decades. It occurs in individuals with fair skin living in warm and sunny climates [1]. Based on cell biology and clinical behavior, SC is classified as melanoma SC (MSC) and non-melanoma SC (NMSC), which is further delineated into basal cell carcinoma (BCC) and squamous cell carcinoma (SCC) [2]. MSC is the most aggressive form of SC [3]. Risk factors of melanoma include intermittent exposure to sunlight, light color of the skin, and geographical location, although one form, acral lentiginous melanoma, occurs in dark-skinned individuals on the hands and feet [4]. Intense exposure to ultraviolet (UV) rays over an extended period of time is the most important modifiable risk factor in the development of NMSC, although ozone depletion, genetics, and immunosuppression may play a role [5]. Indoor tanning is also associated with a significantly increased risk of NMSC and possibly melanoma with a higher risk when below 25 years of age [6]. It is estimated that over 1,00,000 new cases of MSC will be diagnosed in the United States (US) in 2020 and 6,850 people will die from the disease [7]. Early detection of SC minimizes the risk and increases the potential for effective treatment options.

The effectiveness of SC treatment options and prognosis depend on the type of SC, location, size, and subtype of cancer, and the presence or absence of metastases. Treatment options include excisional surgery, topical chemotherapy, radiation therapy, photodynamic therapy (PDT), cryotherapy, targeted therapy, and use of immune response modifiers. Risks and benefits must always be considered. Non-targeted chemotherapy for advanced SC is associated with a variety of organ toxicities. This set the stage for the development of novel targeted therapies. This article summarizes the biology, epidemiology, clinical manifestations, and treatment options for SC and highlights the use of silver nanoparticles (AgNPs) in promising new regimens.

## 2 Methods

A scoping literature search was conducted on PubMed, Google Scholar, and Cancer Registry websites focused on the diagnosis and treatment of SC. Key search terms included “skin cancer, melanoma skin cancer, non-melanoma skin cancer, squamous cell carcinoma, basal cell carcinoma, skin cancer diagnosis, skin cancer prevention, and skin cancer treatment, and silver nanoparticles.” The collected data provided invaluable information about the biology, epidemiology, clinical manifestations, treatment strategies, and therapeutic implications of AgNPs in the management of SC.

## 3 Results and discussions

SC is the most common form of cancer. It affects millions of people worldwide. The most common form of SC is NMSC which encompasses BCC and SCC. Although malignant melanoma is less common (4% of all dermatologic cancers), it accounts for 80% of all SC deaths. A review of the biology, epidemiology, clinical manifestations, and treatment options for SC sets the stage for an appraisal of the molecular mechanism of action AgNP technology as applied to SC management.

### 3.1 SC biology

**Figure 1:**
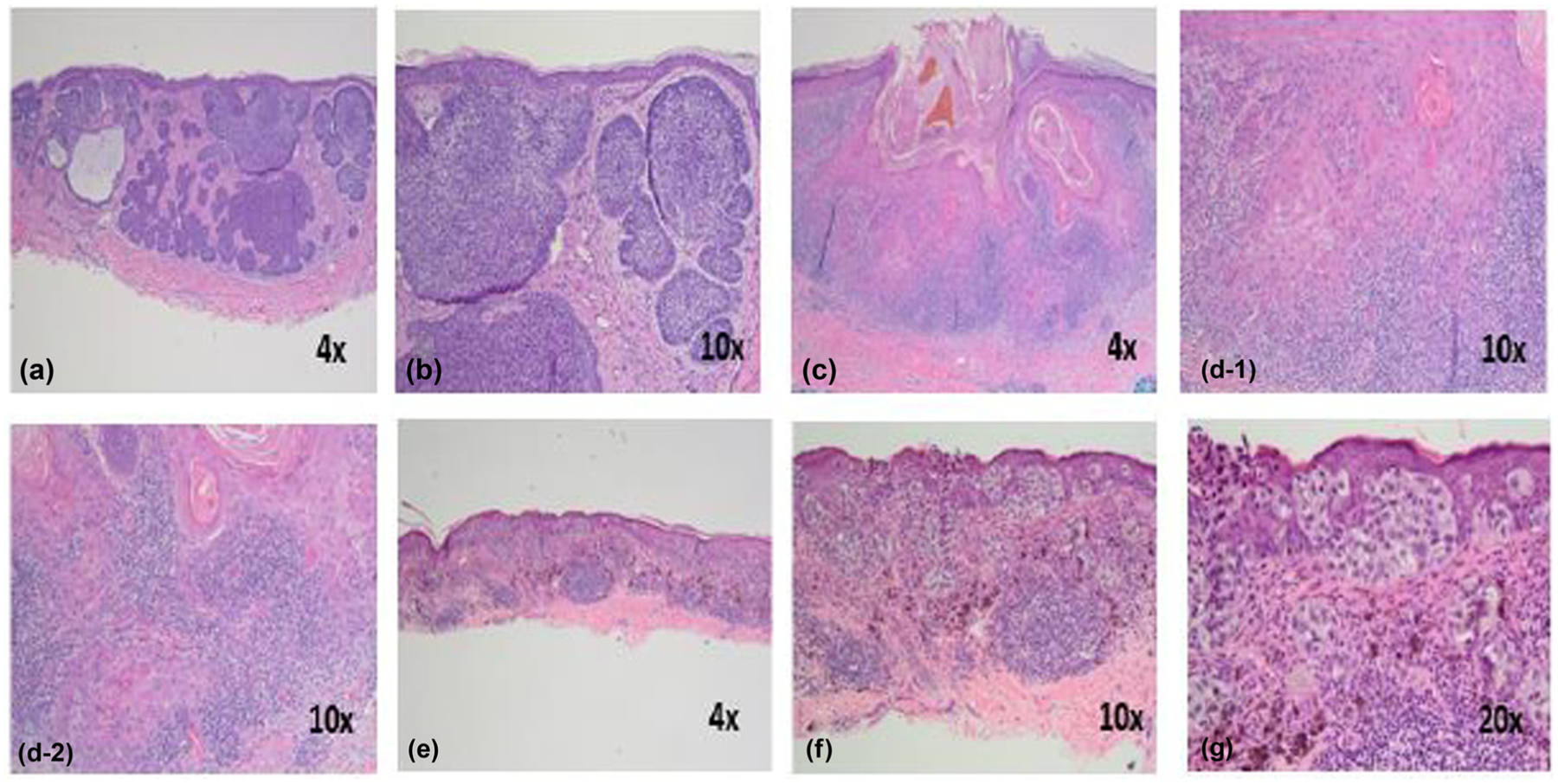
The characteristic histology of different forms of skin cancer: basal cell carcinoma: (a) nodular masses of basaloid cells extend into the dermis with peritumoral stroma (4×); (b) closer magnification highlights the peripheral palisading and tumor retraction artifact (10×); squamous cell carcinoma: (c) scanning magnification highlights a crater-like tumor with irregular masses of atypical keratinocytes invading the dermis. A pronounced surrounding inflammatory infiltrate is seen (4×); (d-1) and (d-2) invading tumor masses with atypical keratinocytes, dyskeratotic cells, and keratin pearls (10×); melanoma: (e) scanning magnification reveals a broad, atypical melanocytic proliferation which is asymmetric and poorly circumscribed (4×); (f) the epidermal proliferation is characterized by variably sized nests at and above the dermoepidermal junction. A robust lymphocytic inflammatory response is present (10×); (g) closer magnification again reveals nests at and above the dermoepidermal junction. The lesional cells are atypical, and single melanocytes are noted at all levels of the epidermis in a pagetoid arrangement (20×) (photographs were provided by Dr. Allison Cruse, Department of Dermatology, University of Mississippi Medical Center, Jackson, Mississippi, USA).

Skin tissue, which is composed of epidermis, dermis, and connective tissue, serves a protective function in mammals. The dermis is composed of nerves, blood vessels, and lymphatic vasculature, embedded in a thick matrix of connective tissue [8]. Skin malignancy occurs when healthy skin cells deviate from their normal function and proliferate in an uncontrollable manner. MSC and NMSC have different cells of origin and specific histology and clinical behavior [2] (Figure 1).

#### 3.1.1 Melanoma

Melanoma, the deadliest form of SC, is initiated in melanocytes. Immunosuppression, sun sensitivity, and exposure to UV radiation in the setting of a genetic predisposition impact the genesis of melanoma [9]. Melanocytes, by function, are pigmented cells responsible for the production of skin and hair color. They are found in the basal layer of epidermis and in hair follicles [10]. Healthy melanocytes secrete melanin causing the tanning response, which absorbs UV radiation and prevents SC [11]. The absence of functional melanocytes in pigmentary disorders such as vitiligo and albinism results in greater susceptibility to the damaging effects of UV radiation [10]. Melanocytes also signal keratinocytes to secrete factors that enhance melanocyte survival, differentiation, proliferation, and motility. Mutations in key growth regulatory genes, secretion of autocrine growth factors, and the loss of adhesion receptors damage intracellular signaling in melanocytes [12]. Therefore, through BRAF (rapidly accelerated fibrosarcoma viral oncogene homolog B) mutation and activation of the mitogen-activated protein kinase (MAPK) pathway, melanocytes proliferate in a manageable fashion to form benign nevi (moles) [11]. Cytological observations of nevi reveal lesions within the cyclin-dependent kinase inhibitor 2A (CDKN2A) and phosphatase and tensin homologue pathways [9]. Further progression through decreased expression of microphthalmia-associated transcription factor leads to the radial-growth-phase MSC [10]. Modulation in expression of the melanocyte-specific gene melastatin 1 (TRPM1) advances the progression into vertical-growth phase cells which form nodules within the dermis which correlate with the metastatic potential of MSC [9]. Other events, including the loss of E-cadherin and increased expression of N-cadherin, αVβ3 integrin, and matrix metalloproteinase 2 (MMP-2), are common mutations in melanoma which reduce apoptosis prolonging the life of these rapidly proliferating melanocytes [9].

#### 3.1.2 Basal cell carcinoma

**Figure 2:**
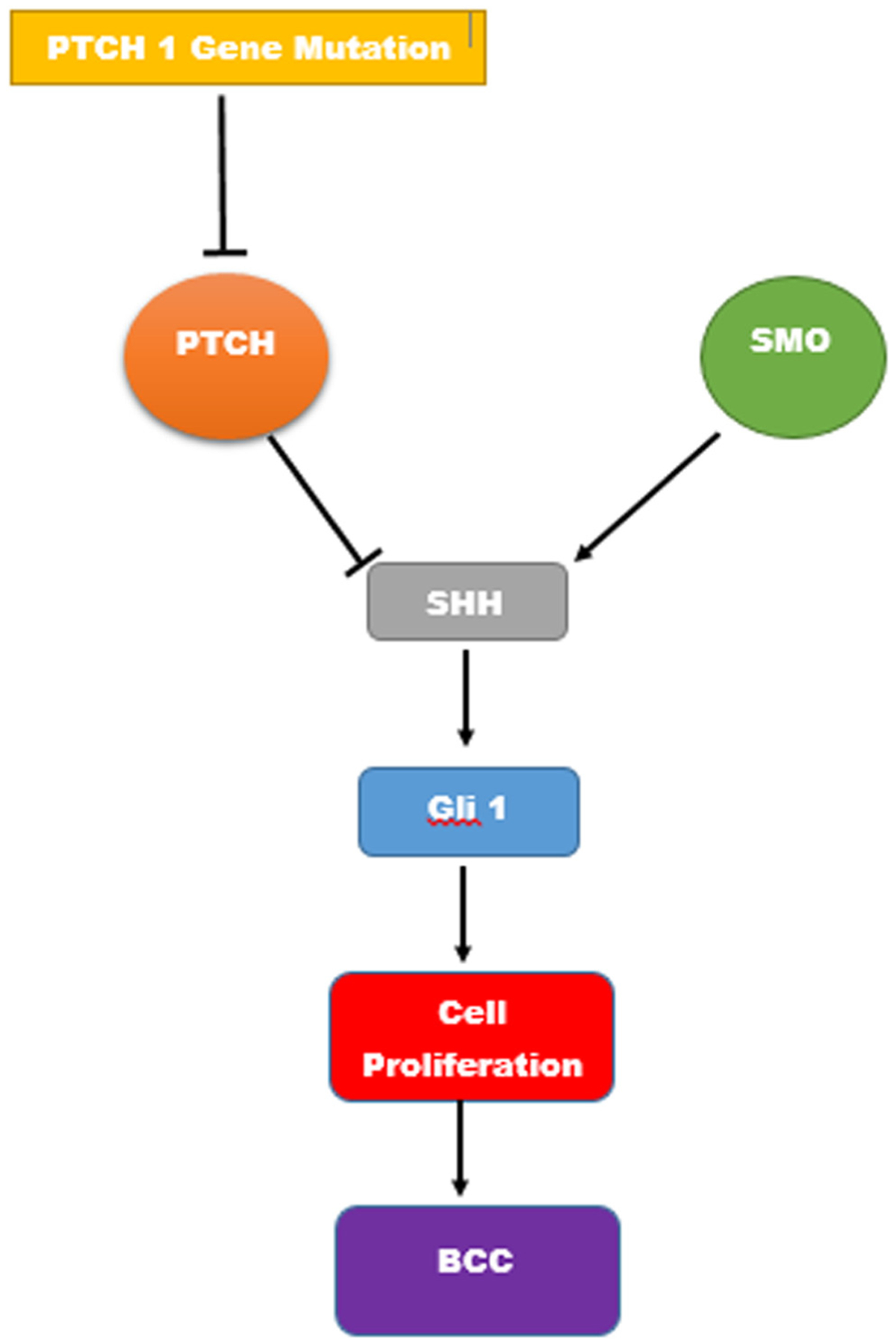
Interactions between Shh, Ptch-1, and Gli-1.

BCC is capable of local tissue destruction and commonly recurs when incompletely excised. Fortunately, it rarely metastasizes. UV exposure from natural sources and tanning parlors is the most important modifiable risk factor in the development of BCC and explains the higher incidence of this tumor in men associated with outdoor occupations and older patients. Genetic conditions may also play a role [5]. Women may be catching up. Between 1973 and 2009, women below the age of 40 years presented a constant linear rise of 6.3% in BCC incidence rates associated with increased occupational and recreational UV light exposure [13]. BCC develops from germi-native cells within the basal layer of the epidermis. Studies showed a differential expression of antigens by BCC, suggesting that tumor cells are proliferative and relatively undifferentiated. Chromosomal abnormalities observed in BCC are associated with invasiveness or histologic subtype of the tumor [14]. Mutations in components of the hedgehog (HH) signaling pathway lead to onset of BCC [15]. Both the autosomal dominant hereditary and non-hereditary forms of BCC are associated with the inactivation of tumor suppressor gene Patched 1 (PTCH1) stationed on chromosome 9q (see Figure 2) [16]. Seven-transmembrane domain G-protein coupled receptor-like protein Smoothened (Smo) is the zinc finger protein (Gli) activator when Ptch is absent. Smo binds to sonic hedgehog (Shh) while activating cytoplasmic Gli transcription factors, thereby triggering an increase in the expression of proteins required for cell proliferation (Figure 2) [15].

#### 3.1.3 Squamous cell carcinoma

SCC is the second most common form of SC with incidence rates doubling in the past three decades [17]. Numerical and structural chromosomal aberrations result from UV exposure in epithelial keratinocytes and contribute to the onset of SCC. Other risk factors include exposure to chemicals, cigarette smoking, and human papilloma virus [18]. UV-irradiated premalignant keratinocytes undergo clonal expansion, developing into actinic keratosis (AK) [19]. Histological studies and molecular evidences have proven that actinic keratoses are precursors of SCC [20]. Uncontrolled proliferation of these keratinocytes in the epidermis produces SCC *in situ*, and dermal invasion is the sine qua non of SCC. Photo-biological studies have demonstrated that UV-R-related mutation accounts for allelic loss on chromosomes, leading to complex genetic alterations such as inactivation of tumor suppressor protein (p53), CDKN2A, amplification of rat sarcoma (RAS) genes, and overexpression of RAS downstream proteins like MAPKs and cyclins [21].

### 3.2 Epidemiology of SC

Skin is the largest organ of the human body, and predictably the most common type of cancer is SC. The number of SC cases has been increasing over the years, perhaps attributing to a combination of factors including longevity, better SC detection, thinning of stratospheric ozone, increased artificial UV exposure at tanning parlors, and increased natural UV exposure at work and at play. The most important modifiable risk factor for skin tumorigenesis is UV radiation [22]. The effect of UV exposure is cumulative in nature as the average age of diagnosis is 65 [7]. MSC is more prevalent in Caucasians than in other ethnic groups (0.1% in Blacks and 0.6% in Hispanics) with a lifetime risk of developing melanoma of about 2.6%. MSC is more common among men; however, the rates are higher in women before the age of 50 [7]. One rare form of MSC, acral lentiginous melanoma, is more commonly associated with dark skin and is not associated with UV exposure [11].

Incidence rates for NMSC are about 18–20 times higher than that of melanoma [23]. During the past 30 years, the worldwide incidence of SCC has been increasing 3–10% per year. During the same period, it is estimated that the BCC incidence rate has risen between 20 and 80% in the US [24]. A Swedish database of family cohort’s analysis revealed that intentional tanning is a contributing factor in SCC incidence in the younger generation [25]. SCC has some association with geographic location as higher incidence rates are observed in tropical regions [26]. In addition, there is a correlation between SC and immunity as patients who received organ transplantation demonstrate a higher incidence of NMSC [27]. Limitations in understanding the incidence of NMSC can be attributed to geographic variability and inadequacy of data from cancer registries.

### 3.3 Clinical manifestations of SC

**Figure 3:**
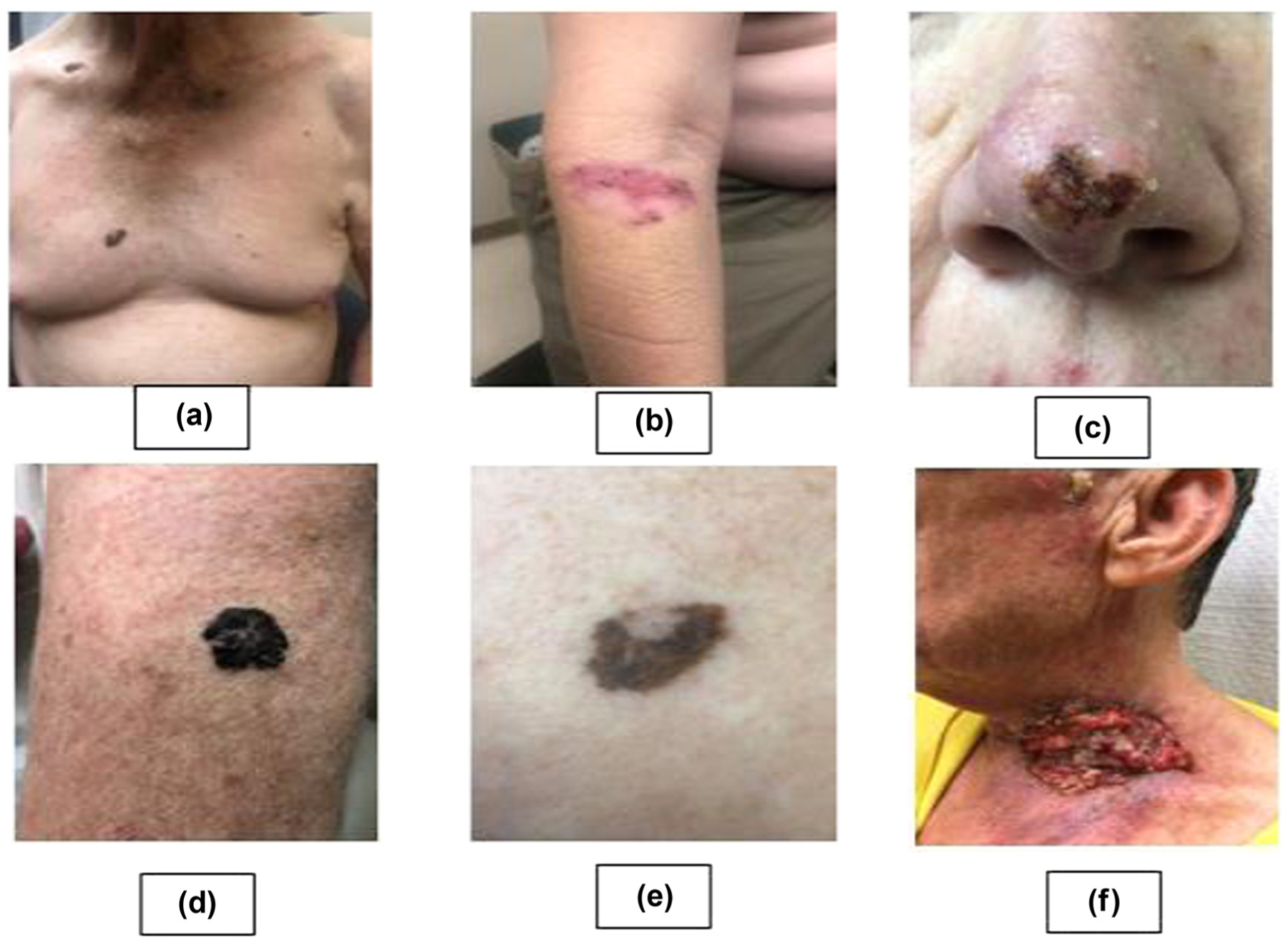
Clinical manifestations of different forms of skin cancer: (a) malignant melanoma distant view; (b) superficial BCC; (c) nodular BCC; (d) melanoma; (e) malignant melanoma with regression; (f) SCC (photographs were provided by Robert T. Brodell, MD, Professor and Chair, Department of Dermatology, University of Mississippi Medical Center, Jackson, Mississippi, USA).

Identification of SC is the most important step for physicians, requiring a careful examination of the entire skin surface. Clinically, skin tumors are diagnosed and prognosis is assessed on the basis of their histopathological appearance (Figure 3).

#### 3.3.1 Melanoma

The diagnosis of primary superficial spreading malignant melanoma is based on the clinical appearance of lesions. A history of recent “change” in a pre-existing nevus or in the appearance of a new pigmented lesion is most important in the early diagnosis of melanoma [28,29]. These features are highlighted in the ABCDE mnemonic for melanoma detection (Asymmetry, irregular Border, variegation of Color, large Diameter greater than 4 mm, and Evolution of change of pigmented lesion) [29]. There is an emphasis on the early diagnosis of cutaneous malignant melanoma since the depth of invasion in the skin and thickness of the lesions are linked to disease prognosis. Malignant melanomas of the skin are commonly found on the back, anterior torso, upper extremities, head and neck in males, and the back, lower legs, upper extremities, and head and neck in females [30]. Melanoma can appear as a raised nodule (nodular melanoma) or flat lesions on the palms of hands, soles of feet, and in the nail bed (acral lentiginous melanoma) [31]. A number of other pigmented lesions can resemble CM either clinically or histopathologically. These include benign juvenile melanoma, blue nevus, halo nevus, giant pigmented nevus, pigmented nevi on palms and soles, and melanotic freckle of Hutchinson [32].

#### 3.3.2 Basal cell carcinoma

BCC lesions are classified clinically as nodular, superficial, and infiltrative based on their growth pattern and degree of differentiation [33]. Clinically, BCC appears as a small, pearly lesion on the face and other sun-exposed areas. As it grows, the tumor outstrips its blood supply and central ulceration and crusting occur. Thus, BCC often is described as the sore that will not heal. Superficial BCC appears as a scaling patch similar to nummular eczema, but these tumors do not itch (see Figure 3a). Larger plaque-like lesions demonstrate numerous pearly papulonodules with telangiectasias at the periphery of a crusted ulcer that has been described as rodent ulcer (see Figure 3b). Cystic nodules may also be present [33]. When BCCs are pigmented, they may mimic seborrheic keratosis or even malignant melanoma [34].

#### 3.3.3 Squamous cell carcinoma

AK is the established precursor for SCC. It appears as 2–6 mm erythematous lesions with a sand-papery scale [19]. When this atypical proliferation of keratinocytes remains in the epidermis, the lesions are termed *in situ*. Bowen disease demonstrates sharply demarcated, erythematous, velvety, or scaly plaques on sun-exposed areas. The other common form of *in situ* SCC, erythroplasia of Queyrat, appears as a red, smooth plaque on the glans penis in uncircumcised men [19]. Invasive SCCs most commonly appear as expanding indurated, crusted, verrucous lesions plaques often with ulceration in sun exposed skin on the head and the neck [35,36] (see Figure 3c). Some SCC lesions are tender, but many are painless. SCC recurrence after treatment is associated with tumor size and factors such as degree of histological differentiation, depth of the lesion, perineural invasion, immune deficiency, and anatomic localization [5]. The lifetime risk of developing SCC in AK patients is 6–10% [37].

### 3.4 Overview of current therapeutic strategies

The optimal treatment option for SC depends on the size, location, and developmental stage of the tumor. Common treatment for larger SCs includes excision, Mohs surgery, or radiation therapy, whereas smaller SCs may be treated with curettage and electrodessication, laser therapy, cryotherapy, or PDT [38].

#### 3.4.1 Excisional surgery

Indolent primary tumors can be treated with elliptical excision. This method involves traditional histopathological processing and “bread-loafing” of the specimen every 1.5–2 mm. This provides a representative view of the tumor margin, but only 1% of the actual margin is viewed [39]. Advantages include histologic verification of tumor margins in representative sections, rapid healing, and the thin scar produced is cosmetically acceptable in the majority of patients. Disadvantages include the risks of hematoma, seroma, infection, and the potential for wound dehiscence [19].

#### 3.4.2 Mohs surgery

Mohs micrographic surgery is a method of surgical excision in which the tumor is removed in stages in an out-patient setting using local anesthesia to ensure that the entire tumor is removed while sparing as much normal tissue as possible. The horizontal frozen sections produced in this manner provide visualization of 100% view of the peripheral and deep margins of each specimen [39]. It is cost-effective in comparison to traditional surgical excision methods [40]. Mohs micrographic surgery minimizes the potential for recurrence in patients with high-risk primary or recurrent BCC or SCC [41]. More recently, the Mohs technique has been used with immunohistochemistry stains to remove selected lesions of malignant melanoma *in situ*, especially those on the face near vital structures [42].

#### 3.4.3 Curettage and electrodessication

Electrodessication and curettage is a technique involving the destruction of tumor and adjacent healthy tissue by cauterization, followed by scraping with a curette [37]. The process is repeated multiple times to increase the probability of complete removal of SC. Nonavailability of specimens for margin evaluation is a drawback [19]. Still, 5-year rates of cure in patients with small primary BCC and SCC are often above 90%. It is, however, not recommended for high-risk tumors [6].

#### 3.4.4 Cryotherapy

Cryotherapy treatment uses liquid nitrogen to freeze primarily BCC and small SCC to tumoricidal temperatures [37]. This avoids complications of bleeding and most often heals in cosmetically acceptable fashion without the line scar typical of an excision. High rates of clearance have been reported [43]. The inability to determine margins and the operator-dependent nature of this process have resulted in this modality being uncommonly used for SC.

#### 3.4.5 Radiation therapy

Radiation therapy is used in older patients with large, aggressive, or recurrent SC who cannot tolerate surgery or in locations where surgical removal is impossible [37]. It produces favorable functional and cosmetic results. It is often used in combination with other therapeutic modalities. High treatment costs, need for multiple visits, and the potential for recurrence of aggressive SC are disadvantages of radiotherapy [40].

#### 3.4.6 Photodynamic therapy

Some superficial BCCs are treated with PDT. The systemic administration of hematoporphyrin derivative or dihematoporphyrin ether followed by irradiation from a tunable dye laser of 630 nm light has largely been supplanted by the topical application of aminolevulinic acid (ALA) followed by blue or red UV light [44]. The mode of action involves absorption of light by the active component – dihematoporphyrin ether generating singlet oxygen, which has cytotoxic effects [45]. Combination of PDT with other topical agents has been studied and found to be effective for superficial SCs [46].

#### 3.4.7 Chemotherapy

Topical chemotherapy with *5-fluorouracil* (5-FU), a structural analog of thymidine that hinders thymidylate synthetase, has been demonstrated to interfere with deoxyribonucleic acid (DNA) synthesis in dividing cells resulting in cell death. This method of treatment is conducive to the prevention and treatment of multiple superficial lesions, such as superficial BCC and SCC *in situ* (Bowen disease), whereas systemic chemotherapy is required for the treatment of larger and thicker tumors [47].

The topical application of retinoids such as 13-cis-retinoic acid has demonstrated positive results with daily application to actinic keratoses and superficial BCC by inducing apoptosis [48]. Retinoids have cytotoxic effects on the growth and differentiation of tumor cells [49]. They also increase in the density of gap junctions between tumor cells twofold and decrease desmosome density by 35% [50].

Studies with non-steroidal anti-inflammatory drugs (NSAID), such as oral and topical *celecoxib* and topical diclofenac, have demonstrated chemopreventive effects by inhibiting angiogenesis and stimulating apoptosis, primarily via cyclooxygenase-2 (COX-2) inhibition [51]. It was shown that levels of COX-2 increased in correlation with the aggressiveness of skin tumors [52].

#### 3.4.8 Targeted immunotherapy

High-dose interleukin (IL)-2, interferon (INF)-α, dacarbazine, carmustine, paclitaxel (taxol), temozolomide, and cisplatin are the most widely used adjuvant immunotherapies for advanced melanoma [9]. Immunotherapy with ipilimumab and the MAPK-targeted inhibitors vemurafenib, dabrafenib, and trametinib are targeted treatment options available for SC patients [11]. Ipilimumab is a Food and Drug Administration (FDA) approved drug for adult patients with metastatic melanoma that acts as an antibody against cytotoxic T-lymphocyte antigen-4 (CTLA-4). It blocks CTLA-4, thereby allowing appropriate T lymphocytes activation. These events restore T-cell proliferation while enhancing the patient’s capacity to survive because of the antitumor immune response [53]. Similarly, the proteasome inhibitor PS-341 has been shown to enhance activity of the drug temozolomide against melanoma [54]. Smoothened (SMO) inhibitors are highly targeted therapies for BCCs. HH signaling pathway, regulated by activating mutations in SMO, is critical in BCC pathogenesis. Itraconazole, sonidegib, and vismodegib are FDA approved drugs that inhibit the HH signaling pathway [55–57]. Chemotherapeutics, such as INF-α and 13-cis-retinoic acid, have been used primarily for SCC in the past [58]. Cemiplumab was recently approved by FDA for the treatment of severe and metastatic SCC [59].

Intralesional injection of IFN has shown promising results in the treatment of NMSC by modulating the development and function of humoral or cell-mediated responses to antigens [60]. Combination therapy of BCC with alfa 2a and 2b has shown to be synergistic through cluster of differentiation 95 (CD95) ligand–CD95 receptor interaction.

*Imiquimod* is another promising topical therapy in the management of select NMSC cases. It promotes immune stimulation by binding to cell surface receptors (toll receptor 7) and stimulating the secretion of cytokines (IL-1, tumor necrosis factor α (TNFα), IL-6, IL-10, and IL-12) producing antitumor effects through mitochondrial mediated apoptosis [61]. The list of some chemical compounds used as biopathway inhibitors for SC treatment is shown in Table 1. The mechanism of action is unknown for some forms of INF treatment as topical immunotherapy. Side effects of flu-like symptoms with one instance of sero-positive rheumatoid arthritis have been reported [62].

Recent studies have proven that epidermal growth factor receptor (EGFR) inhibitors are frequently responsible for cutaneous drug reactions causing an adverse effect on life expectancy, and hence leading to the discontinuation of treatment [63]. It has also been reported that the therapy for larger BCC lesions was less effective as the focus on treating superficial portions of the tumor left a deeper component undetected [64].

#### 3.4.9 Herbal supplements

There is a potential role for dietary and herbal supplements in the prevention and treatment of cutaneous neoplasia. Examples include beta-carotene supplementation, topical combination of vitamin C, vitamin E, and melatonin, low-fat diet, etc. [65,66].

### 3.5 Biomedical applications of nanotechnology in SC treatment

Systemic treatments for skin problems produce potential adverse effects that impact health. Better drug delivery systems that efficiently deliver therapeutic agents through the skin barrier are needed. Optimally, this would be done without chemical enhancers that may damage epidermal cell cohesion and stratum corneum lipids.

Nanotechnology is a blooming area for skin health maintenance, as well as for the diagnosis and management of cutaneous disease. Nanotechnology relies on the interaction at the sub-atomic level with the skin tissue [67]. Drug permeation/penetration is modified via controlled release of active substances by increasing the period of stability on the skin [68], establishing direct contact with the stratum corneum and skin appendages [69], and protecting the drug against chemical or physical instability.

In addition, NPs provide high efficacy, specificity, and cost-effective treatment options [67]. Evidence from research developments in the fields of drug delivery through enhanced skin penetration by NPs is very promising [70]. This is the result of improved methods such as differential stripping, spectrophotometry, and confocal laser scanning microscopy that improve our understanding of intrafollicular drug delivery and kinetics for a variety of drugs across the skin barrier [70].

Nanotechnology research focused on dermatology is ongoing in the development of consumer products (sunscreens, fillers, antimicrobials, and wound care), devices for real-time diagnosis and visualization of tumors, diagnostics for sentinel lymph node assessment, and therapeutic agents including antimicrobials, epicutaneous fillers and paralytics, epidermally localized corticosteroids, gene silencers, epicutaneous vaccines, and inducible therapies activated by optical, magnetic, temperature, and radiofrequency [71].

#### 3.5.1 Drug delivery

The transdermal approach for drug delivery offers the potential for precise targeting of SC [72]. The major challenge is to increase skin permeation of the antineoplastic drug to permit an adequate pharmacological dose. Anticancer drugs that possess hydrophilic properties have a low oil/water partition coefficient, high molecular weights, and ionic characters that impede penetration through the stratum cornea barrier [73]. According to Fick’s second law, the bioactivity of a drug is dependent upon drug permeation, drug concentration in the vehicle, the partition coefficient between the formulation and the stratum corneum, the membrane thickness, and the diffusion coefficient of the drug in the stratum corneum [74]. Nanocarriers such as liposomes, dendrimers, polymersomes, carbon-based NPs, inorganic NPs, and protein-based NPs increase drug concentration in the vehicle and, thereby, increase drug flux [75]. In the following section, several nanocarriers will be discussed with an emphasis on activity against skin neoplasms.

#### 3.5.2 Magnetic NPs

Magnetic NPs are useful tools for theragnostics (the fusion of therapeutic and diagnostic technologies) [76]. Albumin loaded magnetic nanocomposite spheres with 5-FU has been used to treat NMSC [77]. This improves the penetration of 5-FU and also minimizes the side effects of conventional topical drugs. In addition, *in vitro* studies on magnetic nanoemulsion loaded with zinc phthalocyanine showed great potential as synergic application for SC treatment [78]. Cetuximab-coated thermosensitive liposomes loaded with magnetic NPs and doxorubicin have been tested for its efficacy in breast cancer cell-targeted EGFR NP-liposome drug delivery system [79].

#### 3.5.3 Liposomes

Liposomes are one of the most studied nanocarriers for the treatment of cancer [75]. They are spherical shaped, small artificial vesicles synthesized from cholesterol and natural non-toxic phospholipids. Liposomes are promising systems for drug delivery because of their size and hydrophobic and hydrophilic character [80]. Liposomes loaded with doxorubicin [81,82], cisplatin [83,84], oxaliplatin [85], and camptothecin [86] have been used systemically to enhance drugs’ cytotoxicity with minimum side effects. Combinations of topical drugs such as tretinoin and diclo-fenac-loaded liposomes have demonstrated improvement in skin penetration of the drug, over nonliposomal formulations [87,88]. A randomized study of the effect of topical application of liposome encapsulation of T4 endonuclease V lotion in xeroderma pigmentosum represents a new drug delivery approach that transports enzymes through human stratum corneum and presents biologically active proteins into viable epidermis [89]. Thermosensitive betulinic acid-loaded magnetoliposomes showed antitumor activity against aggressive human breast adenocarcinoma cells under hyperthermic conditions [90].

#### 3.5.4 Solid lipid NPs

The NPs, which have been most studied for delivery of topical medications, are solid-lipid NPs and polymeric NPs, synthesized from poly (dl-lactic acid), poly (lactic-co-glycolic acid) (PLGA), and poly-*ε*-caprolactone [91]. Topical application of both solid-lipid NPs and polymeric NPs promotes constant drug release and protection against drug degradation, thereby achieving targeted drug delivery [75]. Application of drugs (doxorubicin) [92], natural compounds (sesamol and resveratrol) [93,94], and photosensitizer (PS) (aluminum chloride phthalocyanine) [95] loaded with solid lipid NPs demonstrates potential for the development of effective SC treatments.

#### 3.5.5 Photodynamic therapy

Topical PDT induces a cytotoxic effect by activation of a PS prodrug, ALA or its methylated ester (methyl amino-levulinate [MAL]), converted by the heme biosynthetic pathway mostly to protoporphyrin IX. Treatment is accomplished by irradiation with a specific UV wavelength in the absorbance spectrum of the drug in the presence of oxygen [75]. This non-surgical treatment method, approved by the FDA, has potential to clear actinic keratoses and superficial BCC and prevent new lesions from occurring. Treatment of a cancerization field in photodamaged skin has been particularly effective in the treatment of NMSC in organ transplant recipients [96]. Recent studies on PSs including topical indocyanine green with indole 3-acetic acid, MAL, have also demonstrated effectiveness [97]. Efforts were made to improve drug penetration and its release through ALA-loaded NPs such as 2-amino-2-deoxy-*β*-D-glucan and succinate-modified chitosan; however, issues were identified with regard to the rate of uptake into neoplastic cells and penetration into tissue [75,98,99].

Other biomaterials include immunolabeled gold nanorods with EGFR antibody inducing apoptosis in a human SCC model using the reactive oxygen species (ROS) mediated mitochondrial pathway under low power laser exposure [100]. Local treatment of BCC patients with 5-FU-loaded polybutyl cyanoacrylate NPs was successful in basal cell tumor resolution [101]. Dietary flavonoids such as apigenin with PLGA (NAp) NPs were effective in treating skin tumors caused by UltraViolet B-rays (UVB) exposure [102]. Nanoencapsulation of apigenin produced better effects because of their smaller size and faster mobility leading to reduction in healthy tissue damage in therapeutic management of SC [75]. Another interesting material is NanoHHI, a polymeric NP-encapsulated hedgehog pathway inhibitor (HPI-1). As discussed earlier, gene mutations in Shh pathway are driving factors for the development of BCC. Small inhibiting molecule specific for Shh signals is under investigation for the BCC-targeted therapy [103].

In conclusion, microparticles are demonstrating efficacy by increasing drug penetration through the skin with improvised drug stability, permitting low skin irritation, and minimizing side effects through drug encapsulation.

### 3.6 Diagnosis

NPs are excited by a white light from a halogen lamp, while a dark field condenser delivers and focuses it on the top of the sample, providing an image of bright object in a dark background with brilliant color depending on the size and shape of the particles. Conjugation of NPs with antibodies by nonspecific adsorption strongly scatters signals facilitating the detection of abnormal growth across weak signal from normal tissue [104].

Gold nanorod might also be used as imaging contrast agents for cancer diagnosis with a conventional optical microscope. Because of high scattering cross-sections and superior photostability of gold (Au) NPs, anti-EGFR-conjugated Au NPs bind specifically to the cancer cells, because of their overexpression on the cytoplasmic membrane of the malignant cells [105,106].

Magnetic NPs enhance targeting with specific cell labeling for early diagnosis of SC [107]. Recent studies revealed a simple and rapid colorimetric detection of melanoma circulating tumor cells using bifunctional magnetic NPs [108]. AgNP-embedded nanoshell structure is used in cancer imaging and photothermal therapy to absorb light and destroy them via photothermal effect [109].

In addition, the field enhances the Raman scattering of adjacent molecules causing a phenomenon called surface-enhanced Raman scattering (SERS) [110]. Because of Raman intensity directly proportional to the square of the field intensity imposed on the molecules, near-infrared-sensitive SERS nanoprobes used for molecular imaging of target cancer cells successfully demonstrated targeting, isolation, and imaging of cancer cells [111]. Through M-SERS conjugated targeting antibodies, the specific cancer cells could easily be isolated by an external magnetic field in a multiple cell population. Application of SERS by gold nanorods to diagnose cancer cells from normal cells specifically bound to human oral cancer cells [112]. Raman tagging is another novel approach where an organic dye molecules with aromatic structures attached to the NPs by physically adsorption or chemically conjugation [113–116]. In addition, multiplexing with SERS labels of Raman reporter molecules is also possible [117].

In light of this, NP-mediated diagnosis appears to be a promising approach for an effective SC treatment by significantly improving detection sensitivity while reducing the signal acquisition time, thereby creating new developments of SERS from bench top to *in vivo* applications and opportunities for further clinical imaging system centered on Raman spectroscopic cancer detection.

### 3.7 AgNP in nanotherapy

AgNPs are the most widely used NPs among all other NPs because of their antibacterial properties [118]. They are high-demand materials for consumer products because of their unique physical and chemical properties. Studies on potential therapeutic implications of AgNPs reveal their wide applications in medicine. AgNPs have been used in medicine, medicinal devices, pharmacology, biotechnology, electronics, engineering, energy, magnetic fields, and also in environmental remediation [119]. Recently, AgNPs have been widely used in healthcare products, the food industry, paints, cosmetics, female hygiene products, medical devices, sunscreen, biosensors, clothing, and electronics [120].

#### 3.7.1 Antibacterial activity of AgNPs

AgNPs are capable of attacking wide variety of pathogens at low concentrations [121]. The multiple antibacterial mechanisms of AgNPs lower the risk of antibiotic resistance. Molecular mechanism of bacterial cytotoxicity involves destruction of cell wall, production of ROS, and damage of DNA [122].

#### 3.7.2 Anticancer effect of AgNPs

Experimental studies have been conducted on the cytotoxicity of AgNPs in different cancers including cervical cancer, breast cancer, lung cancer, hepatocellular carcinoma, nasopharyngeal carcinoma, hepatocellular carcinoma, glioblastoma, colorectal adenocarcinoma, and prostate carcinoma [123]. Contributing factors for effective treatment include dose, time of exposure, and size and shape of the AgNP. Molecular mechanisms of AgNP-mediated apoptosis involve production of ROS, mitochondrial membrane disruption, DNA damage, and signaling pathways leading to programmed cell death [124].

#### 3.7.3 Other medical applications

AgNPs exhibit special physicochemical properties resulting in their wide-spectrum of application in medicine. AgNPs can increase the wound healing rate probably by inducing angiogenesis. Advantages over conventional treatment methods include shorter period and a superior cosmetic appearance, including nearly normal hair growth and less hypertrophic scarring [125]. AgNPs can be used as doping materials for synthetic bone supports [126]. They also play a role in fracture healing as an osteoconductive biomaterial by stimulating proliferation and osteogenic differentiation of mesenchymal stem cells [127]. AgNPs conjugated with polymethyl methacrylate have been used to treat dental dentures through their anitbacterial activity since AgNPs reduce biofilm formation [128]. AgNPs have also been reported to enhance the immunogenicity of vaccines by loading with suitable concentrations of NPs [129]. AgNPs also exhibit anti-diabetic effect via influencing insulin signaling pathway [130].

#### 3.7.4 Biosensing and bioimaging

AgNPs have been extensively applied in various subfields of nanomedicine such as nanoelectronics, diagnostics, molecular imaging, and biomedicine by their enhanced electromagnetic fields [131]. In the following sections, we discussed few applications of nanotechnology in diagnosis of a disease.

##### 3.7.4.1 Plasmonic nanoantennas

AgNPs act as highly sensitive probes beneficial for targeting and imaging of small molecules, DNA, proteins, cells, tissues, and even tumors *in vivo* [109,132,133]. AgNPs behave as nanoscale antennas by enhancing the electromagnetic intensity in its vicinity. SERS technique makes use of the enhanced electromagnetic field, where molecules can be recognized based on their distinctive vibrational modes. SERS helps in the early detection of cancer biomarkers or the detection of drug levels in the blood and other body fluids. M-SERS, along with targeting antibodies, has an ability to specifically target and sort the cancer cells and isolate them by an external magnetic field [131]. This method works by adsorption of molecules on AgNPs where strong field enhancement is generated in the nanogaps for detection to the factor of 10^8^–10^12^ [134]. In a previous study, silica-encapsulated silver-embedded magnetic AgNPs produced stronger SERS signals for targeting breast cancer cells and floating leukemia cells [135]. Using this method, targeted cells can be easily separated from multiple cell population.

##### 3.7.4.2 Nanobiosensor

AgNPs can absorb and scatter light with powerful efficiency. Nanobiosensor is an innovative combination of nanotechnology and optical biosensor finding its application in medical, food safety, environmental monitoring, and drug screening [116,136]. The localized surface plasmon resonance (LSPR)-based nanobiosensor is a new type of optical biosensor technique developed by excitation when the incident photon frequency is resonant with the collective oscillation of the conduction electrons. Recently, LSPR biosensor based on AgNPs for the detection of p53 protein levels from HNSCC patients has been designed [137]. In addition, a combination of triangular plate-shaped AgNPs of different sizes with monoclonal antibodies (mAb) that bind to specific biomarkers acts as a multiplexed lateral flow point-of-care (POC) sensor [138]. AgNPs have become an answer for efforts to develop a POC detection for chronic and acute sickness.

##### 3.7.4.3 Metal-enhanced fluorescence

Metal-enhanced fluorescence is a biotechnology-based tool where metallic nanostructures are used to alter the spectral properties of fluorophores for improved detection in immunoassays, ratiometric sensing, and DNA detection. Regulation in radiative decay rates and resonance energy transfer seems to be the primary role of metal NPs to increase detection sensitivity. Thiolated oligo-nucleotides conjugated to AgNPs on a glass substrate resulted in substantial increase in fluorescence intensity [139]. Enhanced ratiometric fluorescence detection has become possible through silver island films providing up to tenfold increases in fluorescence signal [140]. In addition, metal-enhanced solution assays, fluorescent probes, and planar immunoassays have been proved to show the usefulness of AgNP-enhanced fluorescence [141].

#### 3.7.5 AgNP and SC

With regard to AgNP and SC, their absorption through intact and damaged skin was very low but detectable. However, in case of damaged skin, increased permeation has been observed [142]. In contrast, another study has revealed that AgNPs were able to penetrate through the intact human skin *in vivo* and could be found beyond the stratum corneum at depths of the reticular dermis [143]. The penetration of AgNPs is linked to the size of AgNPs. Human epidermal keratinocytes’ cytoplasmic vacuoles showed the presence of AgNPs (20, 50, and 80 nm) [144], while 100 nm diameter AgNPs were unable to penetrate into the human epithelial line [145] and 0.002–0.02 ppm AgNPs did not penetrate through intact human epidermal keratinocyte cell line (HaCaT) keratinocytes [146].

Dermal and systemic absorption of AgNPs through healthy human skin seems to have different approach. After penetration from intact human skin *in vivo* beyond the stratum corneum, absorbed silver appears as clusters in silver oxide form across the epidermis [143].

It has recently been suggested that AgNPs may increase the rate of wound closure through the initiation of proliferation and migration of keratinocytes and could trigger the differentiation of fibroblasts into myofibro-blasts, thereby promoting wound contraction [147]. The effect of AgNPs on the functionality of repaired skin is through regulation of skin collagen deposition leading to improved tensile properties and better fibril alignments in repaired skin [148]. However, the AgNP-modulated signaling pathway for collagen regeneration is yet to be explored.

AgNPs pretreatment significantly reduced the extent of apoptosis caused by UVB radiation in HaCaT cells as well as induces G1/S phase cell-cycle arrest. Higher internalization of AgNPs in UVB-irradiated cells indicates the involvement of nucleotide excision repair genes in the repair of UVB-induced DNA damage [22].

AgNPs are synthesized by wide range of processes such as physio-chemical, physical, and chemical techniques via appropriate choice of energy source, precursor chemicals, reducing and capping agent, as well as through concentration and molar ratio of chemicals [131]. However, the photochemical synthesis method produces shape and size-controlled AgNPs for conjugating biomolecules such as DNA probes, peptides, and antibodies, which can be beneficial for targeted chemotherapy. This method involves physisorption of the biomolecule on the surface of Ag while irradiating Ag seed solution with a light of selected wavelength [131].

#### 3.7.6 Properties of AgNPs

The physicochemical properties of AgNPs include shape, surface charge and coating, agglomeration, dissolution rate, and LSPR [149]. Previous studies concluded that the electromagnetic, optical, and catalytic properties of AgNPs can be strongly influenced by their size, shape, and distribution, which can often be varied by altering the synthetic methods, reducing agents, and stabilizers [150]. The extent of AgNP cytotoxicity is determined by their surface charge and size. Smaller particles induce greater toxicity because of their larger surface area [151]. Commonly used silver nanostructures in the biomedical field are spherical AgNPs, nano-wires, nanorods, nanoplates, and nanocubes [152]. Different surface charges of AgNP coatings regulate the NP interaction with various biomolecules at the target site [153]. Nano-sized drug formulations regulate skin penetration of the drug depending on design and physicochemical properties of the ingredient [154]. Several studies have focused on the physical and chemical properties of cubosomes in designing anticancer drugs [155]. Therapeutic use of nanodiamonds has been motivated by their potentially advantageous properties such as inertness, small size, and surface structure for labeling and drug delivery [156].

#### 3.7.7 Mechanisms of toxicity of AgNP to cancer cells

**Figure 4:**
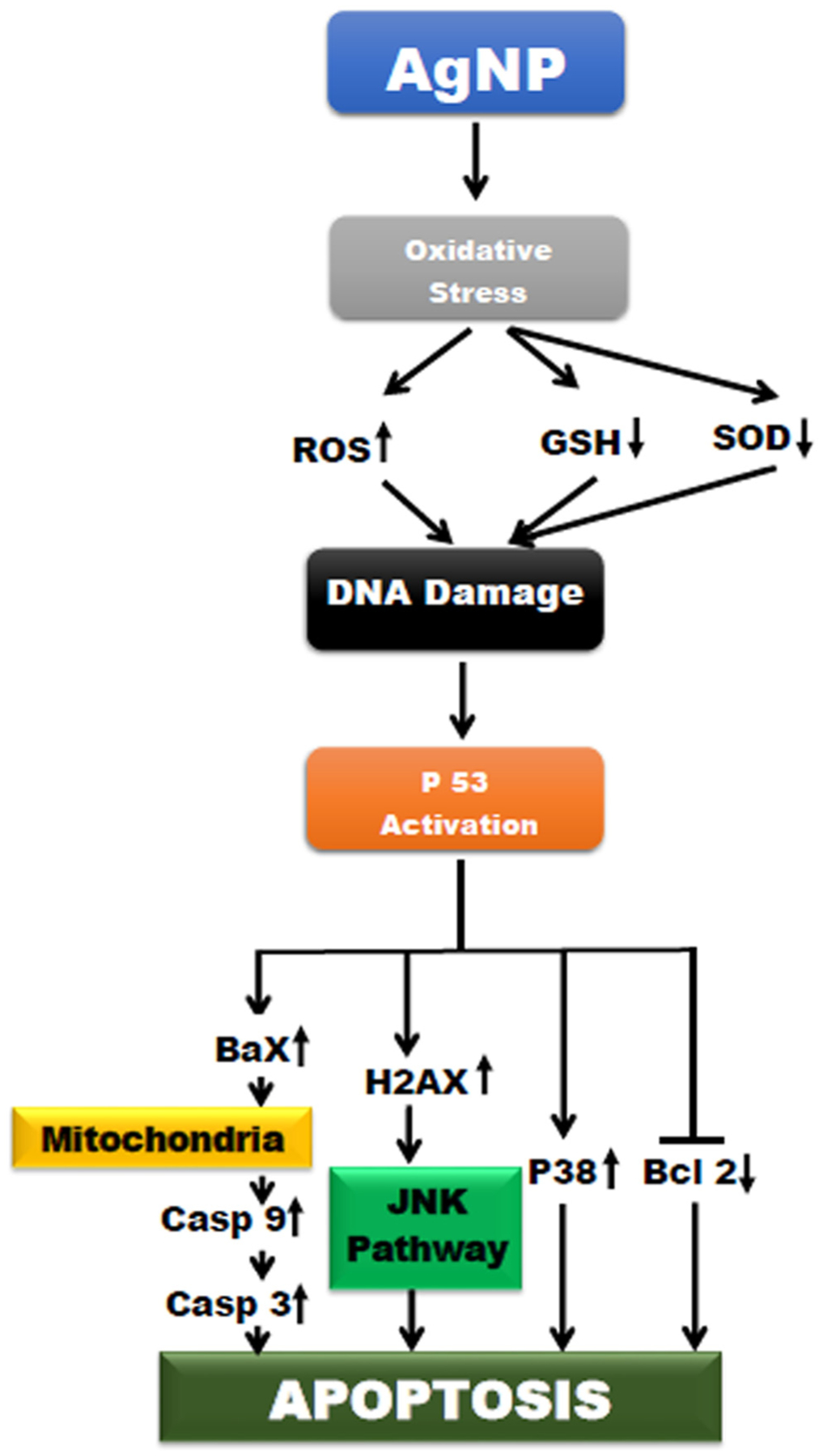
Signaling pathway of AgNP-induced apoptosis.

In spite of a significant research effort focused on the applications of AgNPs, very few studies have been examined on the mechanism of AgNP cytotoxicity. Cytotoxicity depends not only on the NP’s properties but also on the specific organism [157]. The cytotoxic and genotoxic effects of AgNPs depend on the duration, dosage, and temperature along with size, surface coatings, and cell types [158]. The uptake of AgNPs is mainly through endo-cytosis via lysosomes [159]. Exposure to the acidic environment of lysosomes leads to dissolution of AgNPs into Ag ions producing hydroxyl radicals [158]. The internalized AgNPs disrupt the integrity of the cell membrane, causing lysosomal swelling and even rupture lysosomal membranes [160]. The released Ag ions interact with reduced glutathione-S-transferase, superoxide dismutase in the cytoplasm, cell membrane, and inner membranes of a mitochondrion affecting membrane integrity. Furthermore, damage to mitochondria impairs electron transfer, inhibits adenosine triphosphate synthesis, and triggers oxidative stress through lipid peroxidation [161]. AgNPs induce apoptosis through mitochondrial, intrinsic, or p53-mediated pathway [162]. All these events inhibit of cell proliferation through cell-cycle arrest in the G2/M phase [163]. Downregulation of total protein kinase B (AKT) and high expression of p38 are documented along with increased expression of H2A histone family member X (H2AX), Caspase-3, p-p53, and total p53 [164]. AgNP-induced phosphorylation of histone protein leads to activation of c-Jun N-terminal kinase (JNK) pathway [165]. Images from transmission electron microscopy and elemental mapping of single cells have revealed that AgNPs can translocate to the nucleus and cause DNA damage inducing mutations [158] In addition, AgNP can activate a range of pathways such as MAPK and nuclear factor kappa-light-chain-enhancer of activated B (NFƙB) pathways resulting in transcription of many genes involved in the proliferation and inflammatory response [166]. Differential regulation of intracellular factors mediating cell cycle, DNA repair, and inflammation have been associated with AgNP-induced cytotoxicity [167]. A schematic representation of AgNP-induced cytotoxicity is explained in Figure 4 for better understanding of the mechanism.

## 4 Strategies to overcome toxicity

Recognizing that early detection of SC leads to successful treatment with conventional surgical procedures, large tumors, metastatic foci, and tumors near vital structures are best treated with evolving medical therapies, including drug-based chemotherapy, cell-based therapies, and immunotherapy [168,169]. To minimize the side effects of systemic administration of the anticancer pharmaceuticals (e.g., intravenous injections or oral), topical formulations and transdermal alternatives provide novel strategies of drug delivery systems for the effective chemotherapy SC [170].

Although AgNPs induce acute toxic effects to various cultured cells, the toxic effects to normal cells are unclear. The toxicity of AgNPs depends on the particle size, shape, and their surface properties [171–173]. Adverse side-effects can be minimized with polymer–drug conjugates while enhancing drug efficiency, by active or passive targeting of the specific diseased-tissue site [174]. In this section, we briefly discuss the strategies to overcome AgNP toxicity.

### 4.1 Nanocarriers

With the advent of polymer therapeutics, improved solubility of the drug, enhanced bioavailability associated with higher biochemical stability, controlled drug release targeted to specific tissues and organs, and the potential reduction of the total dosage of the drug have been accomplished [13]. Angiogenic niche is the potential site for targeting the nanocarrier [175]. The successful delivery depends on cellular structure of tumor cells, endothelial cells, and disfigured cell shape, along with cell integrity, overcoming immediate clearance from systemic circulation because of engulfing with macrophages and spleen [176,177]. Therefore, physicochemical characterization (particle size, surface charge, density, surface topography) and physiological condition of target site are important for the delivery of nanocarrier [178]. The amount of drug permeated and deposited in skin layers can be altered by adding different kind of nanocarriers such as ethosomes and liposomes to surmount the skin barrier structure and to deliver drugs for the inhibition of UV-induced DNA damage and skin carcinogenesis [179,180]. Therefore, the development of embedded nano-materials such as liposomes, polymer micelles, silicon dioxide, carbon nanotubes, dendritic polymers, gold, silver, and other metal or metal oxides, associated or not with drugs, has been a burgeoning field in recent years [181–183]. Not only AgNP, cerium oxide, and zinc oxide have been extensively studied for melanoma treatment as a recent development in nanomedicine [184]. Surface coating of AgNPs can affect shape, aggregation, and dissolution rate [99]. The type of coating depends on the capping agent properties providing additional functionality [157]. Capping agents seem to enhance the thermodynamic stabilization of NPs by increasing the electrostatic, steric, or electrosteric repulsive forces between NPs, thus preventing their aggregation [185]. Two primary categories of capping agents are organic capping agents (polysaccharides, citrates, polymers, proteins, etc.) and inorganic capping agents (sulfide, chloride, borate, and carbonate) [157]. Polyethylene glycol (PEG)-coated NPs demonstrated stability in highly concentrated salt solutions, whereas lipoic acid-coated particles with carboxyl groups can be used for bioconjugation [131]. Novel study on multidrug resistant tumor cells treated with nanosilver modified with transactivating transcriptional activator (TAT) cell-penetrating peptide showed 24-fold higher toxicity. Same study similarly showed significantly reduced adverse toxicity in a mouse melanoma model [186]. Polystyrene-coated AgNP caused few genetic changes compared to uncoated NPs based on cell viability assay, micronucleus test, and DNA microarray analysis [187]. Thymoquinone PLGA (TQ-PLGA) NPs formulated and characterized using an active compound extracted from *Nigella sativa* – TQ is a biocompatible coating material (TQ-PLGA NPs) with the evaluation of its therapeutic properties in human melanoma cancer cells with 96.8% encapsulation efficiency [188]. Other evidence demonstrates that AgNP coatings interfere with normal healthy skin cells. In a comparative study to understand the behavioral, developmental, and morphological changes on fish treated with AgNPs of different sizes and coatings, it was found that capped AgNPs are less potent than Ag^+^. Therefore, toxicological effects highly depend on particle coating and size, rather than the release of Ag^+^ alone [189]. In addition, citrate, a widely used reducing and capping agent, and PVP-coated AgNPs were tested to be less cytotoxic against human colorectal adenocarcinoma cells [190]. It is noted that surface coating of NP can significantly affect the AgNP-induced cytotoxicity. Alginate and Poly 4-styrene-sulfonic acid-co-maleic acid (PSSMA) capping agents are selectively toxic to the cancer cell line but not to the normal cell line. These results confirm that one of the determining factors for toxicity of AgNPs is the type of capping agent [191]. Nevertheless, the question still remains whether AgNPs can affect keratinocytes and fibroblasts during the healing process.

### 4.2 Green synthesis

Because of the toxic side effects of AgNPs to non-target organs, green synthesis of AgNP has been proposed as a promising technique for SC management. Similar to other synthesis methods, physical characteristics such as size and shape of the NPs are altered by controlling pH and temperature [192]. Green synthesis of AgNPs involves the utilization of bacteria, fungi, yeasts, algae, or plant extracts as reducing and/or stabilizing compounds [157]. Previous studies compared toxicity levels of these green synthesized AgNPs to chemically synthesized synthetic AgNPs [193]. Biosynthesis of AgNPs from different plant parts exhibited cytotoxic effect on skin cells in a dose-response way (Table 2). Several kinds of biosynthesized AgNPs, using bacteria and natural products, have been used in SC. This method of producing ecologically safe NPs reduces the toxic by-products and usage of hazardous chemicals. The benefits of NP biosynthesis include simpler processing methods, shorter synthesis times, high yield, low toxicity, and biocompatibility [194]. Several studies have tested the anticancer effect of green AgNPs on SC. Spherical AgNPs prepared through one-step reaction from *Carpesium cernuum* whole plant extract from reduced silver ions were cytotoxic on *Mus musculus* skin melanoma cells [195]. Dose-dependent antioxidant activity has been observed in skin melanoma cells. A recent study demonstrated that the conjugation between curcumin and silver in nanoform (AgNP-PEG) improved the photostability of curcumin, inducing cytotoxicity on different skin cell lines [196]. Seed extracts of *Trigonella foenum-graecum* have anticancer efficacy against A-431 [197]. Biosynthesized AgNPs of different shapes from Cucurbita maxima (petals), *Moringa oleifera* (leaves), and *Acorus calamus* (rhizome) extracts showed anticancer activity against skin carcinoma [198].

## 5 Conclusions

SC is a prevalent cancer in human populations and is the cause of significant morbidity and mortality. The treatment of both MSC and NMSC includes excisional surgery, Mohs micrographic surgery, curettage and electrodessication, cryotherapy, radiation therapy, PDT, immunotherapy, and chemotherapy, depending on the cancer type, site of occurrence, and patient characteristics. Although standard topical chemotherapy drugs (imiquimod, 5-FU, retinoid, NSAID) and systemic immunotherapy with biopathway inhibitors (vemurafenib, binimetinib, nivolumab, cemiplimab, sonidegib, etc.) have been used widely, the potential side effects of these drugs have led to the development of new therapeutic agents. One approach is the application of nanotechnology in biomedicine, most importantly the evaluation of engineered AgNPs in the diagnosis and management of SC. Research has demonstrated that AgNPs are cytotoxic to SC cells, and their toxicity is mediated through a biochemical mechanism that is triggered by oxidative stress leading to genotoxicity, p53 activation, and apoptosis. The concern over the potential toxicity of AgNPs to normal skin cells led to green synthesis approaches that have been used to produce and test more suitable drugs. The toxicity of these agents is highly dependent upon particle size, shape, and surface properties. There is scientific evidence indicating that the toxicity of NPs to non-target cells can be significantly reduced by modifying their physicochemical properties.

## Figures and Tables

**Table 1:** Chemical compounds used in SC and their biopathway inhibitors’ molecular targets

Inhibitor	Pathway	Gene	References
Vemurafenib	BRAF	RAS/MAPK	[199]
Dabrafenib			[200]
Encorafenib			[201]
Trametinib	MEK	MAPK/ERK	[202]
Cobimetinib			[203]
Binimetinib			[204]
Ipilimumab	CTLA4	Immune Chk	[205]
Nivolumab			[206]
Pembrolizumab	PDLI	PD1	[207]
Cemiplimab			[59]
Itraconazole	SMO	Hedgehog	[56]
Sonidegib			[57]
Vismodegib			[55]

**Table 2:** Green synthesized silver nanoparticles

Plant species	Compound	References
*Euphorbia peplus*	Ingenol mebutate	[208]
*Hypericum perforatum*	Hypericin	[209]
*Coffea arabica*	Coffea	[210]
*Camellia sinensis*	Tea	[211]
*Cucumus longa* Linn.	Curcumin	[212]
*Glycine max*	Genistein	[213]
*Vitis vinifera*	Proanthocyanidin	[214]
*Solanum lycopersicum*	Lycopene	[215]
